# Multifunctional Performance of Hybrid SrFe_12_O_19_/BaTiO_3_/Epoxy Resin Nanocomposites

**DOI:** 10.3390/polym14224817

**Published:** 2022-11-09

**Authors:** Georgia C. Manika, Sevasti Gioti, Aikaterini Sanida, Georgios N. Mathioudakis, Anxhela Abazi, Thanassis Speliotis, Anastasios C. Patsidis, Georgios C. Psarras

**Affiliations:** 1 Smart Materials & Nanodielectrics Laboratory, Department of Materials Science, School of Natural Sciences, University of Patras, 26504 Patras, Greece; 2 Institute of Chemical Engineering Sciences (ICE-HT), Foundation for Research & Technology-Hellas (FORTH), Stadiou Str., Platani, 26504 Patras, Greece; 3 Institute of Nanoscience and Nanotechnology, NCSR “Demokritos”, Aghia Paraskevi, 15310 Athens, Greece

**Keywords:** hybrid nanocomposites, thermomechanical behavior, dielectric properties, magnetic response, multifunctionality

## Abstract

Polymer matrix nanocomposites are widely studied because of the versatility of their physical and mechanical properties. When these properties are present simultaneously, responding at relative stimuli, multifunctional performance is achieved. In this study, hybrid nanocomposites of SrFe_12_O_19_ and BaTiO_3_ ceramic particles dispersed in an epoxy resin matrix were fabricated and characterized. The content of SrFe_12_O_19_ was varying, while the amount of BaTiO_3_ was kept constant. The successful fabrication of the nanocomposites and the fine dispersion of the ceramic particles was verified via the morphological and structural characterization carried out with X-ray Diffraction patterns and Scanning Electron Microscopy images. Dielectric response and related relaxation phenomena were studied by means of Broadband Dielectric Spectroscopy. Dielectric permittivity augments with filler content, while the recorded relaxations, with descending relaxation time, are: (i) interfacial polarization, (ii) glass-to-rubber transition, (iii) intermediate dipolar effect, and (iv) re-orientation of polar-side groups of the main polymer chain. SrFe_12_O_19_ nanoparticles induce magnetic properties to the nanocomposites, which alter with the magnetic filler content. Static and dynamic mechanical response improves with filler content. Thermogravimetric analysis shown that ceramic particles are beneficial to the nanocomposites’ thermal stability. Glass transition temperature, determined via Differential Scanning Calorimetry, was found to slightly vary with filler content, in accordance with the results from dynamic mechanical and dielectric analysis, indicating the effect of interactions occurring between the constituents. Examined systems are suitable for energy storing/retrieving.

## 1. Introduction

Over recent years, the integration of different types of fillers in a polymer matrix has led to the development of high-performance and multifunctional composite materials with a wide range of industrial and technological applications. The incorporation of nanoparticles into polymer matrices enhances their performance in multiple ways. This enhancement depends on the filler’s characteristics, namely its size, aspect ratio, content, and processing conditions. The way the filler is incorporated into the polymer matrix is of paramount importance as it affects the overall dispersion and the subsequent properties of interest. Dispersion is further inhibited by the tendency of the filler particles to cluster in larger agglomerates, something that is mainly driven by the existence of the attractive interactions between the filler particles. Multifunctional materials represent a novel class of engineering materials, which can perform certain operations during exposure to an external stimulus or control signal. The challenge relies on the development of a composite system/device that can execute several functions while being easy to fabricate and remaining cost effective. Novel hybrid nanocomposites could be prepared to address these challenges [1,2,3,4,5,6,7]. It is also of paramount importance to retain their structural integrity and attain a suitable thermal response.

Filled systems are essential for improving and inducing new functional properties otherwise unavailable in neat polymers. For a broad range of applications, suitable fillers can improve the mechanical performance of the matrix and enhance the dielectric, conductive and magnetic properties of the typically insulating polymers. Polymer hybrid nanocomposites can be used for the development of multifunctional and smart engineering systems [1,2,3,4,5,6,7,8,9,10]. Mechanical, electrical, and magnetic responses of these hybrid materials can be controlled by the employed type and amount of the ceramic inclusions. Modern electronic devices require novel high permittivity materials with enhanced dielectric strength operating at a wide temperature range. These materials should exhibit, at the same time, high permittivity and dielectric strength, low loss, improved mechanical performance, ease of processing and a relative low cost. Ceramic particles/polymer composites can be employed in a wide range of applications such as smart skins, acoustic emission sensors, interlayers in ceramic capacitive structures, microwave devices, portable energy storing systems, etc. In addition, by combining ferroelectric and/or piezoelectric and/or ferromagnetic fillers, smart/functional performance can be achieved, due to the varying electric polarization, conductance, and magnetic properties of the ceramic fillers. Smart performance refers to materials that can tune their behavior when responding to an external or internal stimulus. Several properties of these systems can be tuned in a controllable way, such as stiffness, shape, damping capacity, polarization, conductivity, energy storing efficiency, etc. [2,11]. Recently, there have been many studies focusing on the performance of hybrid ceramic particles/polymer composites [9,12,13,14,15,16,17].

Ferroelectric materials such as barium titanate (BaTiO_3_) undergo a disorder to order structural transition at a characteristic temperature (known as the Curie temperature, *T_C_*). Below the Curie temperature, BaTiO_3_ exhibits spontaneous polarization, being at the tetragonal/polar ferroelectric phase [18,19,20,21,22,23,24,25]. At this stage, ceramic barium titanate exhibits domains with randomly oriented spontaneous polarization. Above the characteristic temperature, at the cubic/non-polar paraelectric phase, BaTiO_3_ is characterized by high structural symmetry and the spontaneous polarization is revoked. The application of an electric field upon cooling, while the ferroelectric material is in the paraelectric phase, results in the orientation of the dipole moments of all domains in the direction of the field, when *T* < *T_C_*. This procedure is called poling and the achieved polarization remains even after removing the applied field. Apparently thermally triggered, switching between these two phases leads to systems with tunable performance. Ferromagnetic oxides such as strontium ferrite (SrFe_12_O_19_) are attracting much interest lately due to their magnetic properties, since they have the ability to be magnetized while not being conductive. SrFe_12_O_19_ is a semiconductive material with bandgap ~1.89 eV [26], thus under the influence of an external electric field it performs as dielectric. Therefore, dielectric analysis of such systems could provide additional insight into their electrical properties and molecular structure.

In this work, hybrid nanocomposites were developed by embedding ferroelectric (BaTiO_3_) and magnetic (SrFe_12_O_19_) particles in an epoxy resin. Structural properties, dielectric and thermomechanical response were investigated by employing several experimental techniques. The combination of both fillers allows us to achieve optimal performance, which is mainly related to the synergy of the physical mechanisms of both fillers. BaTiO_3_ induces the functional performance of the transition from the polar ferroelectric phase to the non-polar paraelectric phase, while the SrFe_12_O_19_ induces magnetic properties in the system. The dielectric analysis reveals several relaxation processes that are related to both the polymer matrix (glass-to-rubber transition and re-orientation of polar-side groups) and the presence of the reinforcing phase. High filler loading composites exhibit an intermediate relaxation phenomenon called IDE (Intermediate Dipolar Effect), which is attributed to the intrinsic interfacial polarization phenomena within SrFe_12_O_19_ grains. Strontium ferrite induces magnetic properties to the nanocomposites, which enhance with the magnetic filler content. The presence of ceramic filler strengthens the static and dynamic mechanical response of the nanocomposites, which results in higher thermal stability.

## 2. Materials and Methods

### 2.1. Samples Preparation

A low-viscosity epoxy resin (ER), diglycidyl ether of bisphenol A (DGEBA), with the trade name Epoxol 2004A, and an aromatic amine as curing agent (Epoxol 2004B), obtained from Neotex SA (Athens, Greece), was used as matrix. Spheroid particles of strontium ferrite (SrFe_12_O_19_) with diameter less than 100 nm and barium titanate (BaTiO_3_) with diameter less than 2 μm were employed as fillers. Both reinforcing materials were provided by Sigma-Aldrich (St. Louis, MO, USA). According to the supplier, the densities of the two substances were 5.18 g/mL and 6.08 g/mL, at 25 °C, respectively.

All nanocomposites were prepared using the following procedure: (i) mixing pre-calculated amounts of BaTiO_3_ and SrFe_12_O_19_ into resin prepolymer at ambient conditions, (ii) stirring with a sonicator (Elma S30H, Elmasonic, operating at sweep mode at 280 W) for 10 min at *T* = 50 °C, in order to achieve smooth dispersion of particles in the liquid state, (iii) the mixture was left at room temperature for reducing its temperature and then the curing agent was added in the mixture at a 2:1 *w*/*w* mixing ratio of the epoxy prepolymer and curing agent, (iv) the mixture was sonicated for 10 min at ambient conditions, (v) pouring the mixture into silicon molds and curing at room temperature for 7 days, and (vi) post-curing for 4 h at *T* = 120 °C. The amount of BaTiO_3_ was kept constant at 10 phr (parts per hundred resin per mass) in all fabricated composites, while the concentration of SrFe_12_O_19_ was a varying parameter taking the values: 5, 10, 15, 20, 40 and 50 phr. The amount of BaTiO_3_ was selected by the evaluation of previous studies [22,27,28]. For comparison reasons, the pure epoxy resin specimen was also prepared.

### 2.2. Scanning Electron Microscopy

For the evaluation of the nanocomposite’s morphology, the possible presence of agglomerates and the quality of dispersion of the reinforcing phases within the epoxy matrix, images of electron microscopy were taken by means of Scanning Electron Microscopy (SEM) EVO MA 10 Zeiss apparatus.

### 2.3. X-ray Diffraction (XRD)

For the XRD analysis, a Bruker AXS D8 Advance device with a Bragg–Bretano geometry was employed. More specifically, a LynxEye detector and Cu Ka spectral line (λ = 1.54062 Å) were used as incident radiation. The slit of the source was 0.6 mm and the current voltage sources were set at 40 kV and 40 mA, respectively. Finally, the scan mode was continuous with an angle step of 0.02° (2θ) and the scan speed was 0.5 s/step.

### 2.4. Broadband Dielectric Spectroscopy (BDS)

All specimens were electrically characterized via BDS. The frequency range was varied from 0.1 Hz to 10 MHz via an Alpha-N-Frequency Response Analyzer, which was supplied by Novocontrol Technologies. The temperature was controlled by Phecos system (Novocontrol Technologies, Montabaur, Germany) with ±0.1 °C accuracy, while the applied *V_rms_* was constant at 1 V. A two gold-plated dielectric system (BDS 1200) was employed as a dielectric cell. Isothermal frequency scans were performed for every sample from 30 °C to 160 °C with a temperature step of 5 °C. The real-time acquisition of all data was conducted via WinDeta software (Novocontrol Technologies). All dielectric measurements were conducted according to the ASTM D150 specifications.

### 2.5. Magnetic Measurements

The magnetic response of the fabricated systems was investigated via a Vibrating Sample Magnetometer (VSM, Princeton Applied Research) at ambient temperature. The applied magnetic fields ranged between 20 and 20 kOe.

### 2.6. Thermomechanical Response

The thermal response of all fabricated systems was examined via Differential Scanning Calorimetry (DSC) and Thermogravimetric Analysis (TGA) via the TA Q200 and TA Q500 devices, both provided by TA Instruments. DSC thermographs were assessed in the range from 20 to 100 °C with a ramp of 5 °C/min. TGA tests were conducted from ambient temperature to 600 °C with a heating rate of 10 °C/min. Static mechanical tests were performed with an Instron 5582 Universal Testing Machine in tension, at room temperature with a 5 mm/min rate. Dynamic Mechanical Analysis (DMA) experiments were conducted via a TA Q800 device (TA Instruments, New Castle, DE, USA), in the temperature range from ambient to 100 °C with a heating rate of 5 °C/min at 1 Hz dynamic excitation. The employed type of test was three-point bending.

### 2.7. Energy Storing and Retrieving

Fabricated systems were imposed in DC charging tests followed by discharging in the absence of applied voltage. During the tests the time-dependent charging and discharging currents were recorded and then exploited in evaluating the stored and the recovered amounts of energy. Tests were conducted by means of a High-Resistance Meter, DC (Agilent 4339B) according to the ASTM D257 specifications. All tests were carried out at room temperature and the charging voltages were 50, 100, and 150 V. The under-test specimens were placed in a parallel plate capacitor and charged for 60 s. After charging, and removing the applied voltage, the discharge current was recorded in real time. Prior to all tests, a discharging short-circuit procedure was followed in order to eliminate the presence of pre-stored charges in the samples [10].

## 3. Results

### 3.1. Morphological/Structural Characterization

**Figure 1:**
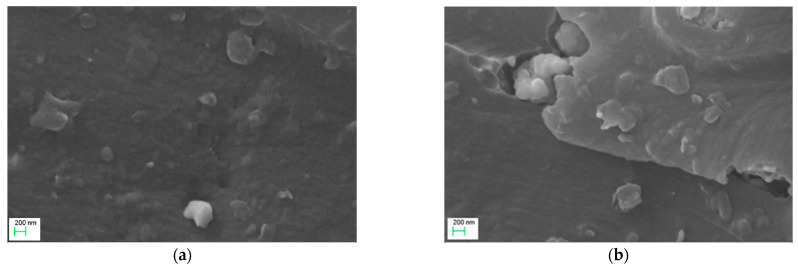
SEM images for the: (**a**) 5 phr SrFe_12_O_19_/10 phr BaTiO_3_/epoxy and (**b**) 50 phr SrFe_12_O_19_/10 phr BaTiO_3_/epoxy, nanocomposites.

**Figure 2:**
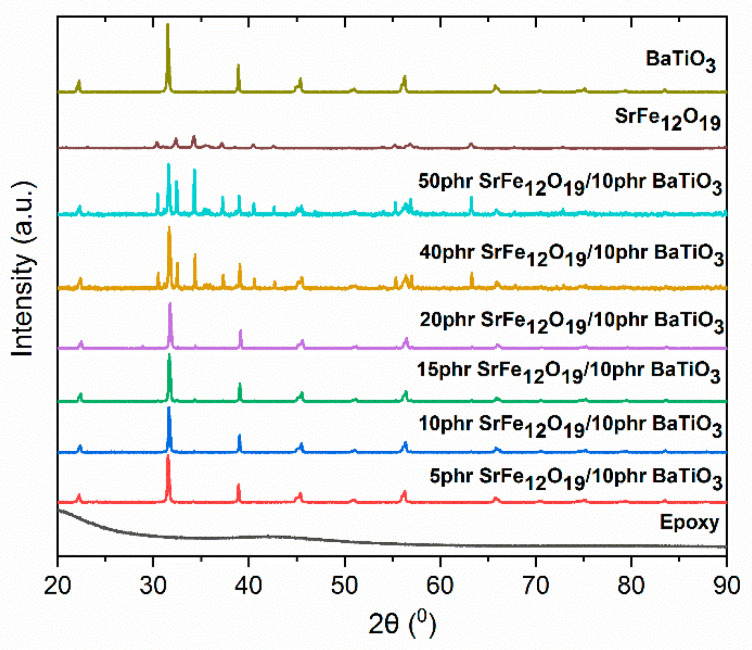
XRD patterns of all studied systems and SrFe_12_O_19_, BaTiO_3_ powders.

SEM imaging of ER with 5 phr SrFe_12_O_19_/10 phr BaTiO_3_ and 50 phr SrFe_12_O_19_/10 phr BaTiO_3_ filler content are shown in Figure 1a,b, respectively. The cryo-fractured surface of the studied samples indicates a fine dispersion of the reinforcing phases in the polymer matrix without extensive aggregates, even at high filler loading. XRD patterns of all hybrid systems are presented in Figure 2.

Since the epoxy resin has no diffraction peaks due to its amorphous state, all recorded peaks are characteristic for the tetragonal structure of BaTiO_3_, as well as for the structure of SrFe_12_O_19_. Specifically, nanocomposites patterns include the (100), (101), (111), (002), (200), (210) and (211) diffraction peaks occurring at 22.2, 31.5, 38.9, 44.9, 45.4, 51.0, and 56.3° of the 2θ, respectively, being characteristic for the BaTiO_3_ particles. The characteristic peaks of the tetragonal phase (002) and (200) are present in the patterns of all nanocomposites. In addition, characteristic peaks at 30.4, 32.4, 34.2, 37.1, 40.4, 42.6, and 63.2° of 2θ, corresponding to (110), (107), (114), (203), (205), (206), and (220) planes, respectively, provide evidence for the embedded SrFe_12_O_19_ particles and their hexagonal crystal structure [29]. It is apparent that the XRD patterns of all hybrid systems include peaks originating from both fillers, while peak intensity increases analogically with filler content. XRD patterns denote the successful incorporation of fillers in the polymer matrix.

### 3.2. Dielectric Characterization

**Figure 3:**
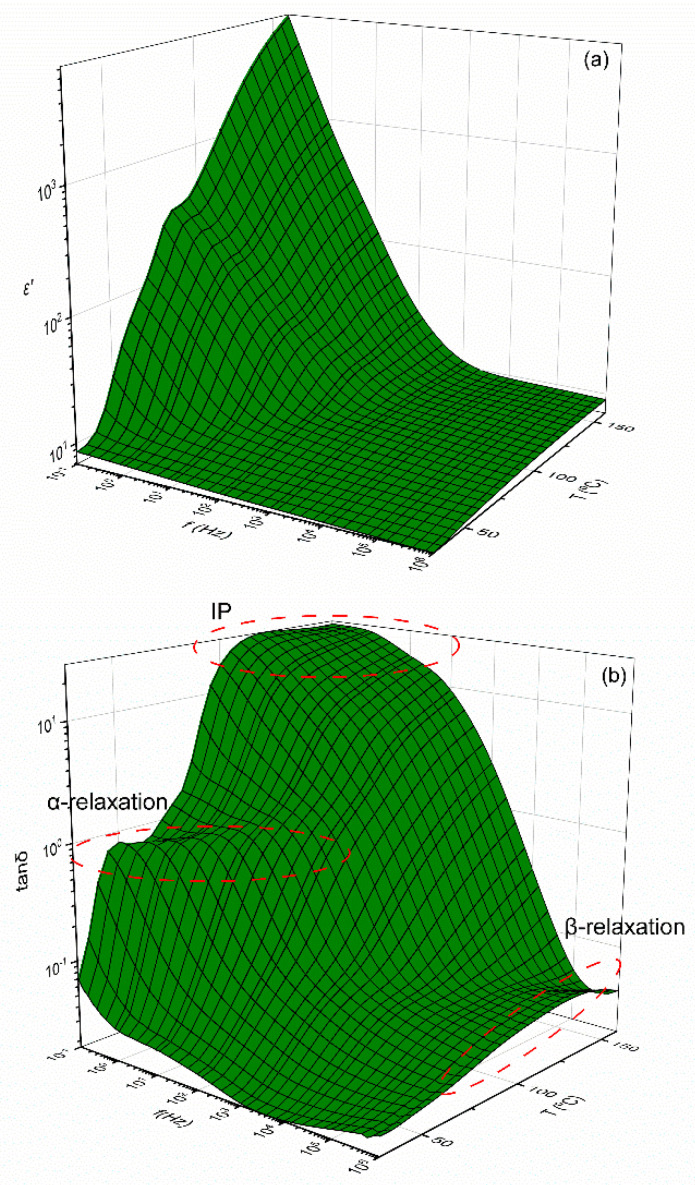

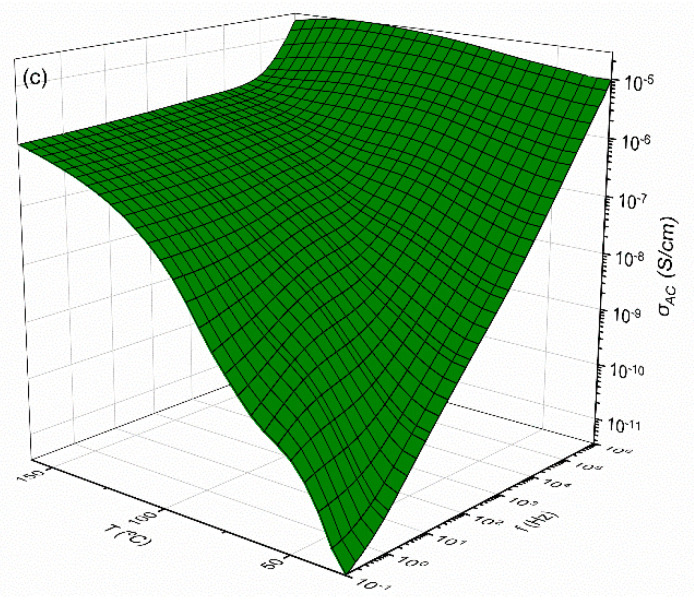
Dielectric spectra of 10 phr SrFe_12_O_19_/10 phr BaTiO_3_/epoxy nanocomposite as a function of temperature and frequency for the: (**a**) real part of dielectric permittivity, (*ε*′), (**b**) loss tangent (tan*δ*) and (**c**) AC conductivity (*σ_AC_*).

The electrical properties of polymer matrix composites are related to dielectric relaxation phenomena occurring under AC conditions. These relaxation phenomena are associated with the dipolar orientation effects of both permanent and induced dipoles and in some cases to the space charge migration. The presence of the reinforcing phases affects all relaxation processes in multiple ways; thus, it is crucial to examine the influence of the fillers upon these relaxation processes. Furthermore, 3D dielectric spectra of the real part of dielectric permittivity, loss tangent and AC conductivity, as functions of temperature and frequency, are presented for the 10 phr SrFe_12_O_19_/10 phr BaTiO_3_/epoxy nanocomposite in Figure 3a–c, respectively.

Figure 3a indicates that the real part of dielectric permittivity (*ε*′) diminishes with the increase of frequency. At low frequencies, dipoles attain sufficient time to orient themselves in the direction of the AC applied field, leading to higher *ε*′ values. At high frequencies, dipoles fail to be aligned in the direction of the AC field, resulting in low polarization level and *ε*′ values. In addition, temperature facilitates the polarization process because of the thermal agitation of dipoles, thus *ε’* acquire high values at low frequencies and high temperatures. Figure 3b shows the formation of three peaks, indicating the presence of relaxation processes. At intermediate frequencies and temperatures, a strong relaxation process appears, which is related to the glass-to-rubber transition of the amorphous epoxy resin (α-relaxation). At high frequencies, the observed relaxation process is associated with the re-orientation of the polar-side groups of the main polymer chains (β-relaxation). In addition, in the low frequency and high temperature range, Interfacial Polarization (IP), also referred to as the Maxwell–Wagner–Sillars effect (MWS), is present. The physical origin of IP is related to the existence of unbounded charges, from the stage of the composites’ fabrication, which accumulate at the interface of the constituents, forming large dipoles with enhanced inertia to the applied field. IP is characterized by long relaxation times and for this reason occurs at high temperatures and low frequencies [11,30]. Figure 3c exhibits the variation in *σ_AC_* upon temperature and frequency. It is obvious that at low frequencies the influence of temperature is more pronounced, revealing a thermally activated conduction mechanism. It seems that the influence of temperature decreases upon frequency, and at high frequencies *σ_AC_* appears to be temperature independent. At a constant temperature, *σ_AC_* follows Equation (1):

(1) $$\sigma_{AC}\left(\omega \right)=\sigma_{DC}+A{\left(\omega \right)}^{s}$$

where *σ_DC_* is the *ω* → 0 limiting value of the *σ_AC_* (*ω*) and *A*, *s* are the parameters depending on temperature and filler content. Equation (1) is also called “The AC universality law’’ due to its general validity [31,32].

## 4. Discussion

There are several formalisms (i.e., dielectric permittivity, electric modulus, AC conductivity and complex impedance) that can be employed for the analysis of dielectric relaxation phenomena. All four formalisms can be used for the description of complex dielectric phenomena, which are present in polymer composites. However, there are cases in which one of them can be more suitable to reveal and analyze a physical mechanism. In the present study, the formalisms of dielectric permittivity, electric modulus, and AC conductivity were employed for the interpretation of dielectric data. Electric modulus is very effective in eliminating the parasitic effect of electrode polarization [33,34,35]. Electric modulus is defined as the inverse quantity of complex permittivity (Equation (2)):

(2) $$M^{*}=\frac{1}{\varepsilon^{*}}=\frac{\varepsilon^{\prime }}{\varepsilon^{\prime 2}+\varepsilon^{\prime \prime 2}}+i\frac{\varepsilon^{\prime \prime }}{\varepsilon^{\prime 2}+\varepsilon^{\prime \prime 2}}=M^{\prime }+iM\prime \prime$$

where *ε*′, *ε*″, *Μ*′ and *Μ*″ are the real and imaginary part of dielectric permittivity and electric modulus, respectively.

**Figure 4:**
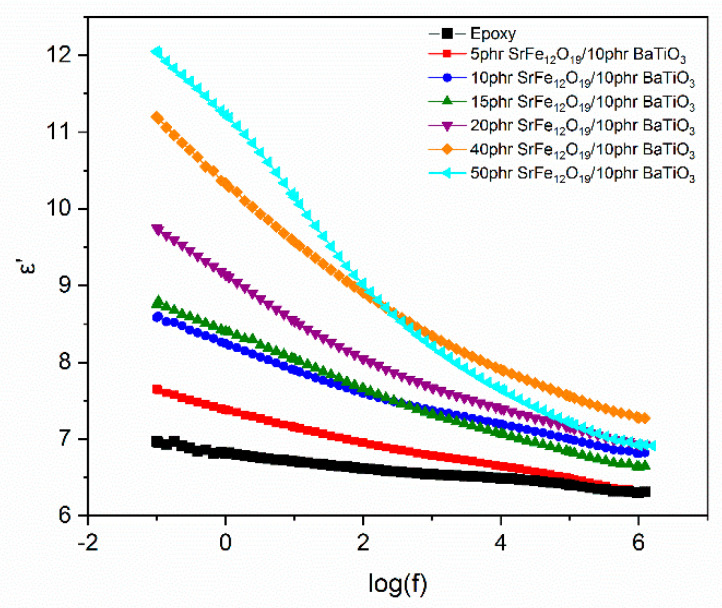
Real part of dielectric permittivity as a function of frequency, at 30 °C, for all studied systems.

The dielectric reinforcing ability of the employed fillers is shown in Figure 4, where the real part of dielectric permittivity as a function of frequency, at 30 °C, for all studied systems is depicted. Hybrid systems acquire a higher value of *ε*′ compared to the epoxy resin in the whole frequency and temperature range.

**Figure 5:**
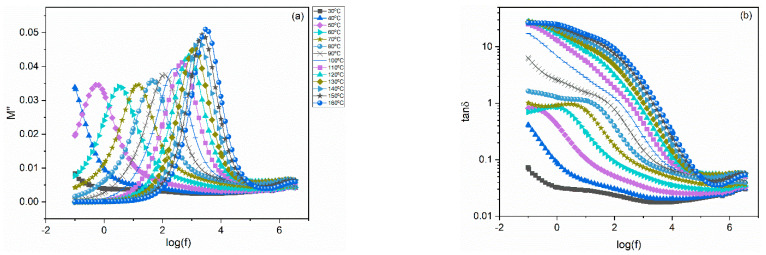
Imaginary part of electric modulus (**a**) and loss tangent (**b**) versus frequency for the 10 phr SrFe_12_O_19_/10 phr BaTiO_3_/epoxy nanocomposite. Notation of temperature is the same in both graphs.

**Figure 6:**
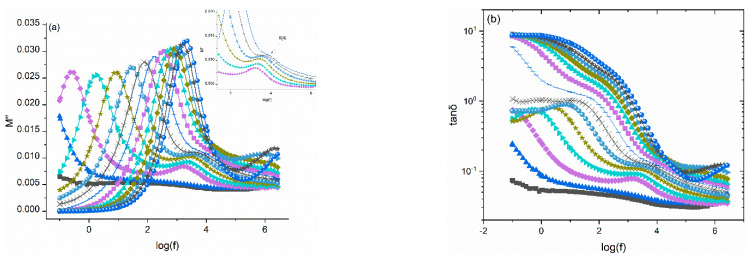
Imaginary part of electric modulus (**a**) and loss tangent (**b**) versus frequency for the 40 phr SrFe_12_O_19_/10 phr BaTiO_3_/epoxy nanocomposite. Notation of temperature is the same with Figure 5.

Figure 5 and Figure 6 present the frequency dependence of the imaginary part of electric modulus and loss tangent at various temperatures for the 10 phr SrFe_12_O_19_/10 phr BaTiO_3_/epoxy and 40 phr SrFe_12_O_19_/10 phr BaTiO_3_/epoxy nanocomposites, respectively. The presence of all relaxation processes becomes evident through the formation of loss peaks in the corresponding plots of *M*″ and tan*δ*. In all samples, at least two relaxation processes are evident: the α-relaxation process in the intermediate frequency range, and at high frequencies the β-mode is derived. It is very interesting to note that in high filler-loaded systems another relaxation process appears in the frequency range between the α and β-mode. This process is absent in the spectra of pure ER and low filler-loaded systems. Since this relaxation is observed between the slow α-mode process and the fast β-mode, it is referred to as the Intermediate Dipolar Effect (IDE), shown in Figure 6 [36]. IDE is attributed to the polarization effects taking place between grains of the ceramic fillers (SrFe_12_O_19_), which relax under the influence of the alternating electric field. IDE has been observed in other ceramic particles/polymer composites and it has been attributed to intrinsic interfacial polarization of the ceramic domains [36,37,38,39,40,41]. Previous studies [22,40] have shown the absence of IDE from the dielectric spectra of BaTiO_3_/epoxy nanocomposites, and thus it is assigned to the strontium ferrite. Inside the grains of polycrystalline SrFe_12_O_19_ a number of charge carriers and possibly defects migrate under thermal activation. Their motion is restricted by potential barriers between adjacent crystal domains and thus they are accumulated at the interior interfaces, resulting in polarization effects. The occurring process is an intrinsic interfacial relaxation in the ceramic grains, and it cannot be confused with the IP (MWS) effect, which is present in heterogeneous systems and arises from the polarization effects at the boundaries (interface) between the various phases.

**Figure 7:**
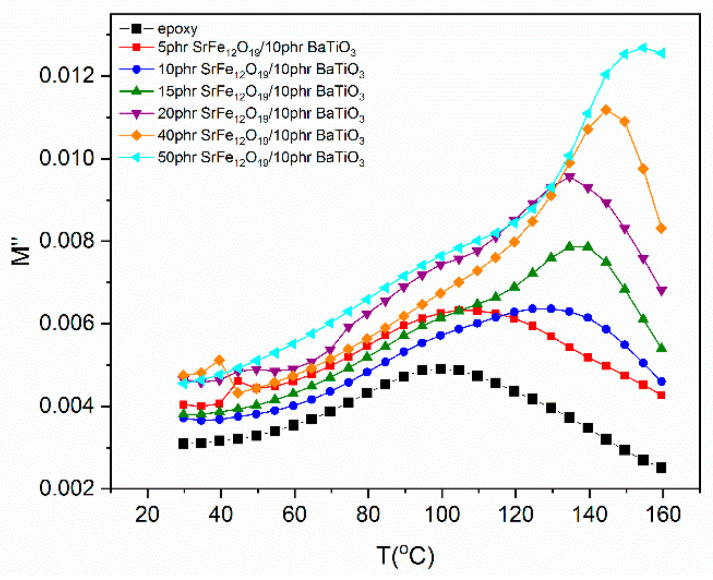
Imaginary part of electric modulus versus temperature for all systems at 1 MHz.

The variation of modulus loss index with temperature, at 1 MHz, is depicted in Figure 7. The broad peak recorded at intermediate temperatures is assigned to β-relaxation mode and linked to the re-orientation of the polar-side groups of the polymer chains. In addition, a second peak is also observed at higher temperatures as the SrFe_12_O_19_ concentration increases. This peak is related to the IDE process, and it is evident in all nanocomposites with SrFe_12_O_19_ loading higher than 5 phr. Processes with higher relaxation times (i.e., α-relaxation and IP) are not shown in Figure 7, since their peaks are formed at higher frequencies, because of the frequency–temperature superposition outside of the “window” of observation.

**Figure 8:**
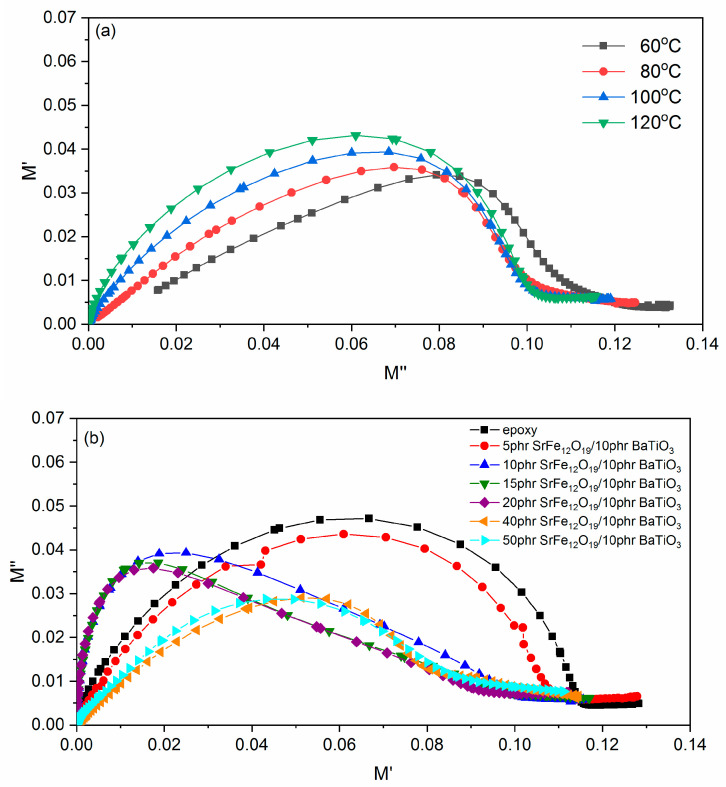
Cole–Cole plots for the: (**a**) 10 phr SrFe_12_O_19_/10 phr BaTiO_3_/epoxy nanocomposite at several temperatures and (**b**) for all examined systems at 100 °C.

Cole–Cole plots for the 10 phr SrFe_12_O_19_/10 phr BaTiO_3_/epoxy nanocomposite at various temperatures are presented in Figure 8a, while in Figure 8b Cole–Cole plots at 100 °C of all studied systems are shown. In the Cole–Cole presentation, relaxation mechanisms become evident via the formation of completed or even uncompleted semicircles. In the case of pure Debye processes, that is, relaxations characterized by a single relaxation time, perfect semicircles are formed with their center lying on the x-axis [11,30,33]. However, it is well known that dielectric relaxations in polymers and polymer composites deviate significantly from pure Debye behavior, because of the distribution of relaxation times [11,30]. In condensed matter, such as polymer-based materials, interactions between polar and/or charged entities occur, leading to processes corresponding to symmetrical or non-symmetrical distribution of relaxation times, which could also superimpose [11,30,42]. In Figure 8a, α- and β-relaxation are shown. Formed curves deviate significantly from a perfect Debye process. The main process, indicated by the oblate semicircles, is attributed to the glass-to-rubber transition, followed by a weak tendency of forming a second semicircle assigned to β-relaxation. Although difficult to be observed, a slight change in the slope of the curves at the low-frequency edge could be considered as arising from the IP process. With the increase of temperature, data coincide at the origin of the graph, denoting that no other process is expected at lower frequencies [33]. The area of the oblate semicircles seems to augment with temperature, representing the resulting increase in the capacitance of the system.

**Figure 9:**
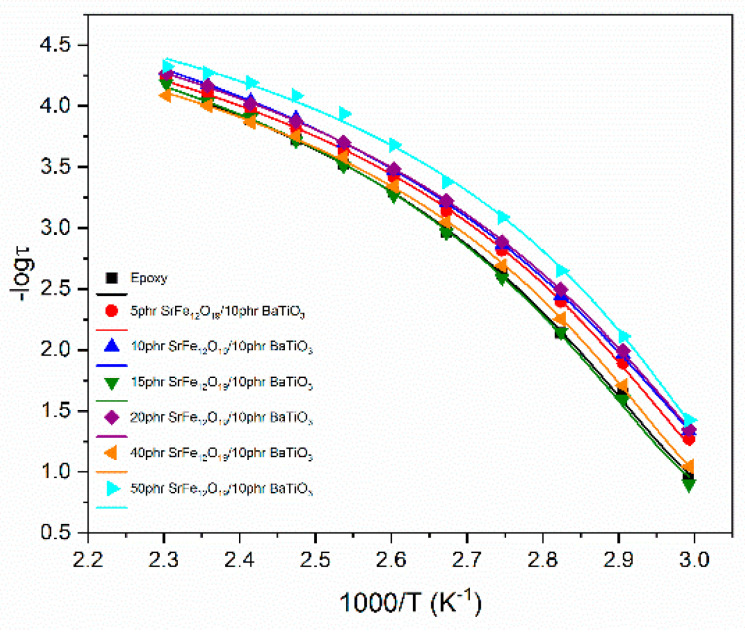
Relaxation time as a function of reciprocal temperature for α-relaxation process.

**Figure 10:**
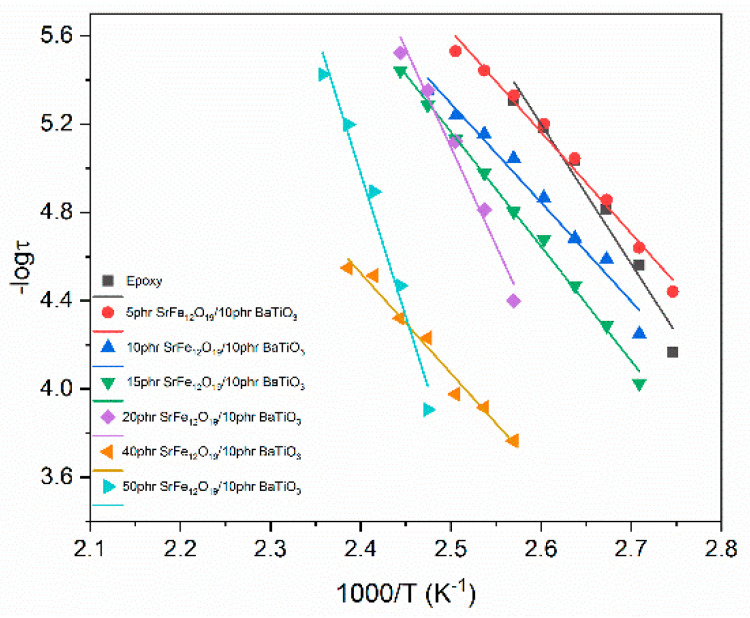
Relaxation time as a function of reciprocal temperature for β-relaxation process.

**Figure 11:**
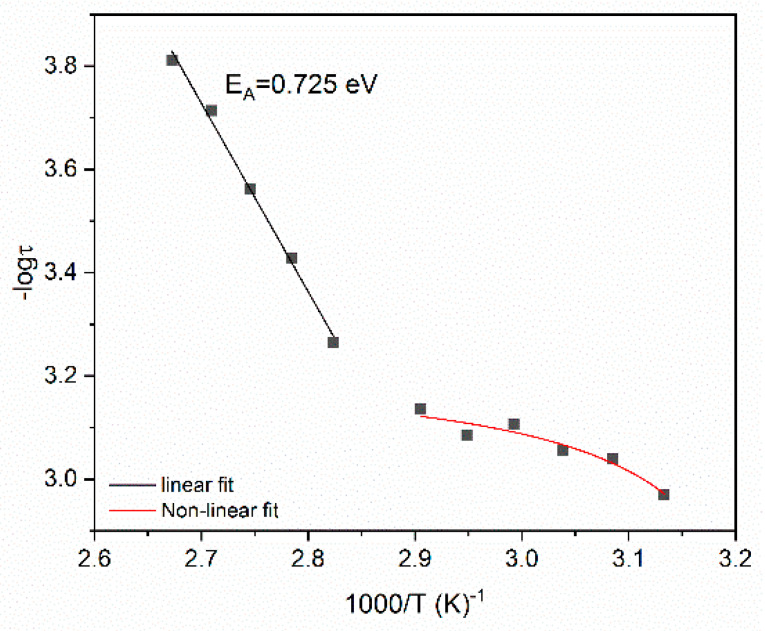
Relaxation time as a function of reciprocal temperature for the IDE process in the 40 phr SrFe_12_O_19_/10 phr BaTiO_3_/epoxy nanocomposite.

Relaxation dynamics can be studied by plotting the relaxation time as a function of reciprocal temperature for each system and for every relaxation process. The relaxation dynamics of all studied processes are presented in Figure 9, Figure 10 and Figure 11. The β-relaxation and the IDE process can be described by the Arrhenius-type temperature dependence of Equation (3):

(3) $$\tau_{max}=\tau_{0}\exp\left(\frac{E_{A}}{k_{B}T}\right)$$

where $\tau_{0}$ is a pre-exponential factor expressing the relaxation time at very high temperature and is considered temperature independent, and *E_A_* is the activation energy of the process. A semilogarithmic plot of relaxation time versus (1000/*T*) can be used for the determination of *E_A_*. Although IP follows an Arrhenius dependence on temperature, a limited number of loss peaks could be extracted from the recorded data, leading to a non-reliable fitting procedure, and for this reason it is not presented here.

On the other hand, α-mode exhibits a Vogel–Fulcher–Tammann behavior described by Equation (4):

(4) $$\tau_{max}=\tau_{0}\exp\left(\frac{AT_{0}}{T-T_{0}}\right)$$

where *A* is a parameter that is a measure of activation energy and *T*_0_ is the Vogel temperature (also known as ideal glass transition temperature) [11,30,42]. In the Supplementary Material the detailed fitting procedure employed for the determination of the loss peak points for the data presented in Figure 9, Figure 10 and Figure 11 is given. It is worth mentioning that in the case of β-mode and IDE relaxation the Havriliak–Negami model was used for the fittings, while in the case of α-relaxation the frequency of the loss peaks was extracted from the *M*″ versus logf plots.

Determined values for the α-mode and β-mode via fitting data with Equations (3) and (4) are listed in Table 1.

Dynamics for the α-relaxation process follow the VFT equation, as described above, since relaxation rate increases rapidly with increasing temperature because of the reduction of free volume. Glass-to-rubber transition is influenced by the presence of both fillers inside the polymer matrix and specifically by the particle–particle and the particle–polymer interactions. Table 1 indicates that *T*_0_ slightly decreases at low SrFe_12_O_19_ concentrations compared to epoxy. The 15 phr SrFe_12_O_19_/10 phr BaTiO_3_/epoxy and the 40 phr SrFe_12_O_19_/10 phr BaTiO_3_/epoxy nanocomposites acquire the highest *T*_0_ values. Parameter *A* increases upon the addition of SrFe_12_O_19_ at low to moderate concentrations (5 to 20 phr) indicating that the process is delayed, probably due to strong interaction among macromolecules and both types of particles. Then, a significant reduction of *A* is observed at the higher SrFe_12_O_19_ loadings, implying that the process is facilitated. The decrease of the *A* parameter indicates that particle–particle interaction of both types of fillers is dominant, thus facilitating the cooperative relaxation of the polymer chains. Obtained results are in accordance with the discussion of the effect of filler loading derived from the Cole–Cole plots of Figure 8.

Activation energy reflects the occurring interactions within the nanocomposites and is a measure of the potential barriers exerted by the dipoles’ environment to their orientation. For the β-relaxation, *E_A_* initially decreases upon the addition of SrFe_12_O_19_ filler in the low filler loadings. This finding shows that nano-inclusions facilitate the orientation of the polar-side groups of the polymer matrix at low content, since particles are apart from each other and interact strongly with the main chains. In general, activation energy increases with filler content, because particles become closer and anchor on the main polymer chain. In the case of the 40 phr SrFe_12_O_19_/10 phr BaTiO_3_/epoxy system, the lowering of *E_A_* is attributed to the strong interactions between particles, which dominate upon macromolecule/particle interaction and do not exert spatial restrictions, since the generated clusters are small and scarce. Further increase in filler loading, causes an increment of activation energy, which reaches its highest value for the 50 phr SrFe_12_O_19_/10 phr BaTiO_3_/epoxy nanocomposite. The latter indicates that the presence of both fillers obstructs the orientation of the polar-side groups, due to spatial restrictions related to the formation of clusters, because of the strong interactions between SrFe_12_O_19_ and BaTiO_3_ particles.

Figure 11 indicates that IDE follows an unusual evolution with temperature. For the temperature range of 75–100 °C, it follows an Arrhenius-like behavior, while at a lower temperature range (45–70 °C), it follows a VFT behavior. The intrinsic interfacial polarization phenomena within SrFe_12_O_19_ particles are responsible for this response. At high temperatures, IDE, as an interfacial polarization effect, follows a typical Arrhenius behavior. However, at lower temperatures it seems that IDE is influenced by α-relaxation. The influence of α-relaxation upon IDE has been reported previously [38] and has been attributed to the proximity of the temperature ranges where the two processes occur. The latter becomes evident by comparing the temperature ranges of Figure 9 and Figure 11. The value of activation energy obtained for its high-temperature part is 0.725 eV for the nanocomposite with 40 phr SrFe_12_O_19_, which is close to relative values of hybrid systems [40] and deviates from values of binary composites reinforced with ZnO or TiO_2_ particles [38,41]. The activation energy of IDE should be related to the type of filler (i.e., crystal structure) and the size of domains within the ceramic grains.

**Figure 12:**
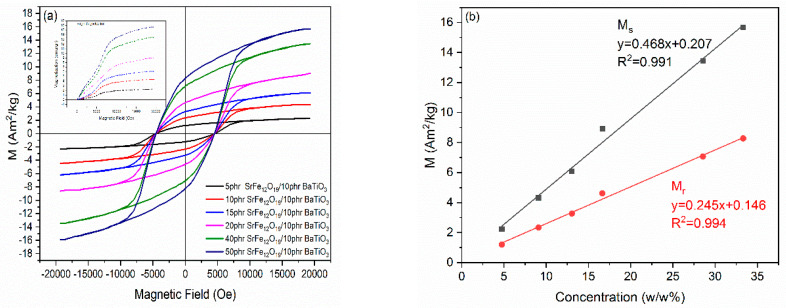
(**a**) Magnetic hysteresis loops for the nanocomposites with varying SrFe_12_O_19_ content. (**b**) The variation of magnetic saturation (*M_s_*) and magnetic remanence (*M_r_*) as a function of magnetic filler content.

The magnetic hysteresis loops of the nanocomposites, at ambient temperature, are shown in Figure 12a. The ferromagnetic behavior of strontium ferrite is induced into the nanocomposites and their magnetization increases with magnetic filler content, which is lower from the corresponding value of the monolithic SrFe_12_O_19_ because of the presence of the two non-magnetic phases (i.e., epoxy resin and BaTiO_3_). Coercivity attains constant values in all nanocomposites (~4.5 kOe), indicating that the coercive field is an intrinsic property of the employed magnetic phase [43]. The inset of Figure 12a shows the virgin magnetization curves of the nanocomposites. From the slope of their linear parts at low magnetic fields and by taking account of the strontium ferrite density, magnetic susceptibility of nanocomposites can be determined. Obtained values are listed in Table S1, and as expected, increase with filler content. Magnetic saturation (*M_s_*) and magnetic remanence (*M_r_*) of the nanocomposites as a function of magnetic phase content are shown in Figure 12b. The observed linear relation of magnetization upon SrFe_12_O_19_ content can be considered as an indication for the fine dispersion of magnetic nanoparticles [9,43] and provides the effectiveness to tailor the magnetic response of the nanocomposites by controlling the amount of the employed magnetic nanoparticles.

**Figure 13:**
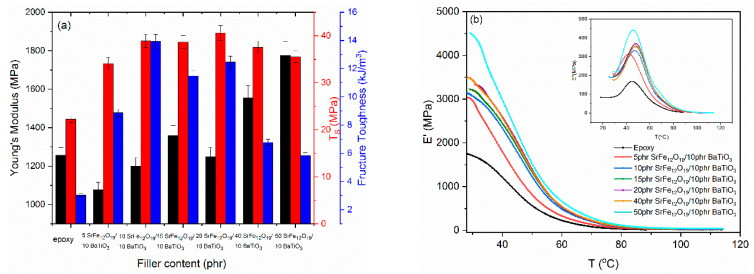
(**a**) Young’s modulus, tensile strength, and fracture toughness for all studied systems versus filler content. (**b**) Storage modulus and loss modulus (inset) as a function of temperature for all studied systems.

Static mechanical properties were investigated via tensile tests at ambient temperatures. Figure 13a summarizes the results. Young’s modulus initially decreases with filler content, which is afterwards followed by an augmentative response with SrFe_12_O_19_ nanoparticle content. Stiffness of nanocomposites appears to enhance with SrFe_12_O_19_ nanoparticles, providing an indirect indication for their good wetting and strong adhesion with the polymer matrix. Tensile strength and fracture toughness are also favorably influenced by the presence of fillers. Tensile strength increases significantly with respect to the unfilled matrix and retains almost constant values up to the system with the highest reinforcing phase content. Fracture toughness, which is an expression of the system ductility, increases up to the 10 phr SrFe_12_O_19_/10 phr BaTiO_3_/epoxy nanocomposite. At higher concentrations, it follows a diminishing tendency that is more pronounced at the two higher SrFe_12_O_19_ concentrations. The possible existence of small clusters can be considered as responsible for this detrimental effect, since clusters and aggregates act as stress-raisers within the nanocomposites. However, from Figure 13a it is evident that the systems’ mechanical integrity is improved by the presence of ceramic particles.

The dynamic mechanical response of the examined systems is depicted in Figure 13b. Storage modulus increases with filler content in the glassy state, denoting the strengthening ability of the reinforcing phases. A step-like transition is observed in the temperature range of 40 to 60 °C, which is indicative of the glass-to-rubber transition of the thermosetting matrix. The transition range shifts to higher temperatures with filler content, denoting the good adhesion between the reinforcing particles and the matrix. The inset of Figure 13b presents the variation of loss modulus with temperature for the same systems.

TGA thermographs were employed for studying the thermal degradation of the fabricated systems. In their spectra, two mass loss mechanisms are included, shown in Figure S2.

The first process appears between 150 and 250 °C and is assigned probably to the breakdown of unreacted epoxy rings and to the possible existence of impurities. The second process related to the decomposition of the epoxy matrix is recorded between 300 and 400 °C. Nanocomposites’ thermal stability improves with ceramic particles because the first degradation mechanism shifts towards higher temperatures. The temperature where the 5% initial mass loss of the examined systems appears is listed in Table S1.

**Figure 14:**
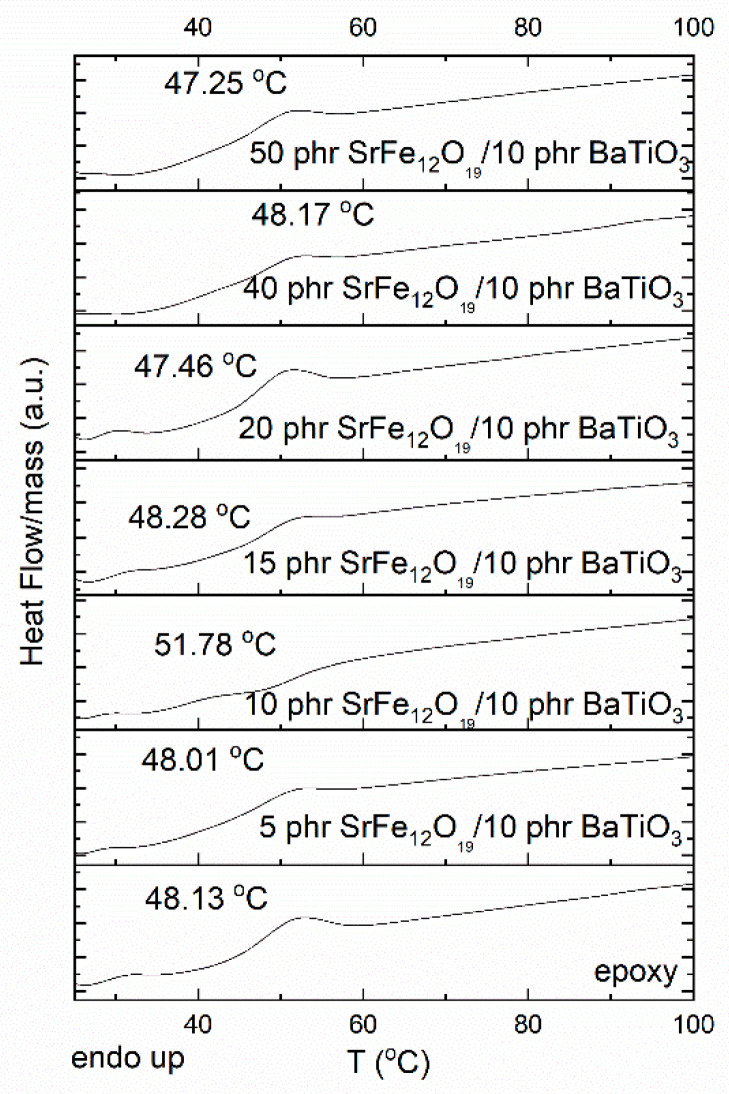
DSC thermographs for all the studied systems.

Finally, DSC graphs were used for studying the thermal events occurring in the examined systems from ambient temperature up to 100 °C. Since the epoxy matrix is an amorphous thermosetting polymer and the reinforcing phases are ceramic crystalline particles with very high thermal stability, no other effect is expected to occur in this region besides the endothermic increase of specific heat capacity. The step-like increase of specific heat capacity corresponds to the glass-to-rubber transition of the matrix and can be exploited for the determination of glass transition temperature (*T_g_*). Glass transition temperature was evaluated via the point of inflexion of the step-like increase of specific heat capacity via suitable software provided by TA Instruments. Figure 14 presents the DSC thermographs of all examined systems. The determined values, which do not vary significantly with filler content, are listed in Table S1. It is well known that the values determined for *T_g_* via different experimental techniques do not coincide [10,11,12]. However, the recorded variation tendency with filler content is in agreement with the previously discussed dielectric results.

**Figure 15:**
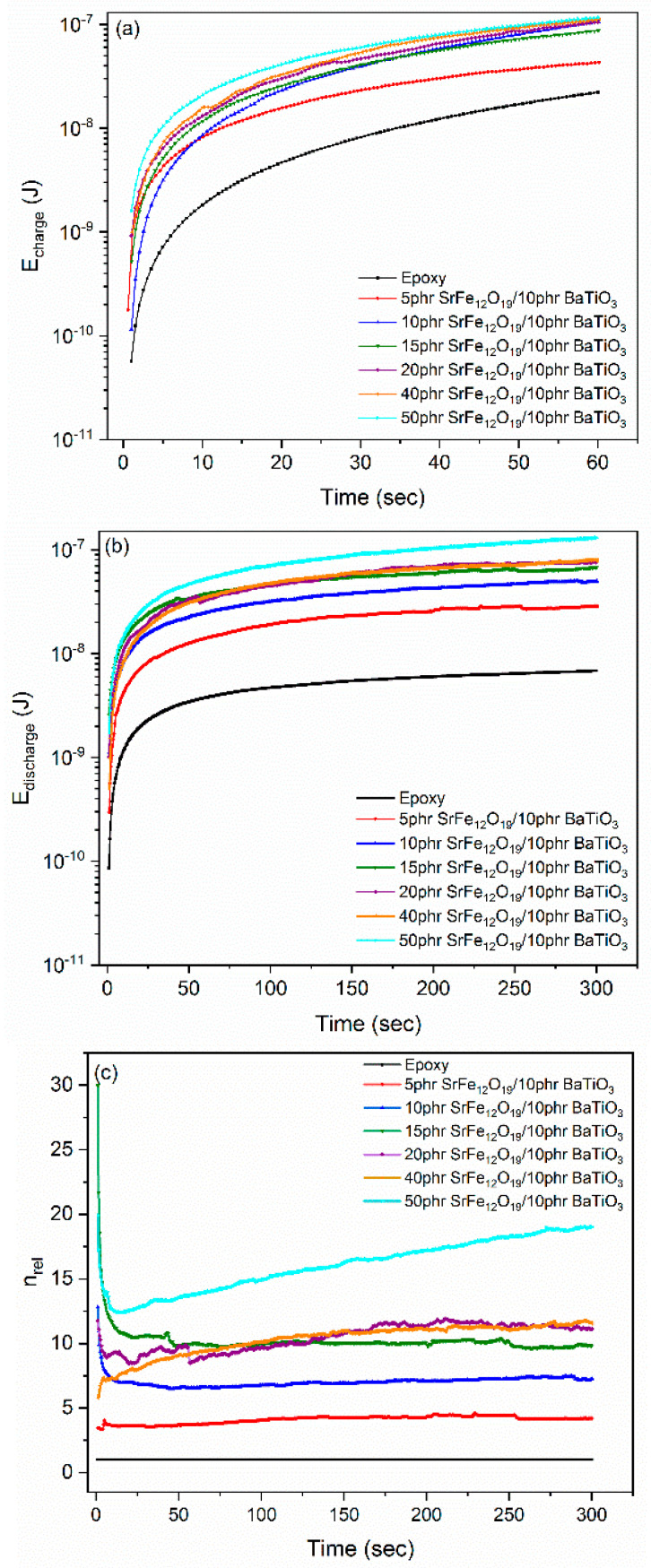
(**a**) Charging (storing) energy, (**b**) discharging (retrieving) energy, and (**c**) the relative retrieved energy, as a function of time at room temperature.

The stored and retrieved energies were evaluated by integrating the time-dependent charging/discharging current functions, via Equation (5):

(5) $$E=\frac{1}{2}\frac{Q^{2}}{C}=\frac{1}{2}\frac{{\left[\int I\left(t\right)dt\right]}^{2}}{C}$$

where *E* is the stored or retrieved energy at the nanocomposite, *Q* is the amount of charge, *I*(*t*) is the charging or discharging current and *C* is the capacitance of the composite as derived by the BDS measurements [10]. In Figure 15a,b, the stored and retrieved energies for all studied systems and for charging voltage 100 V are presented as a function of time. Both energies increase with filler content since the integrated ceramic inclusions are acting as a distributed network of capacitors.

The applied voltage forces the charge carriers to migrate within the nanocomposites. However, the insulating matrix exerts potential barriers, diminishing the charge migration. Temperature could provide sufficient energy to carriers to overcome the barriers, and thus conductivity increases. At room temperature, a limited number of charges has the ability to overcome the potential barriers, so charge migration is restricted, and conductivity attains low values. Charges are accumulating at the phases’ interface and trapped. The application of external voltage causes a reduction of the potential barriers, and charges could migrate through the interfacial area executing a trapping/detrapping sequence. The relative coefficient of energy efficiency can be calculated via Equation (6):

(6) $$n_{rel}=\frac{E_{retr,\;comp}}{E_{retr,\;matrix}}$$

where *E_retr,comp_* and *E_retr,matrix_* are the recovered energies from a nanocomposite and the matrix under the same charging voltage, respectively. Figure 15c depicts the relative coefficient of energy efficiency as a function of time for the examined systems at 100 V charging voltage. Obviously, the ability of restoring energy is enhanced with filler loading.

From the presented results and the conducted analysis, it is apparent that the fabricated hybrid nanocomposites exhibit multifunctional performance since they simultaneously acquire improved dielectric, thermomechanical, and magnetic properties, and have energy storing/retrieving ability.

## 5. Conclusions

Hybrid SrFe_12_O_19_/BaTiO_3_/epoxy nanocomposites were fabricated and studied varying the SrFe_12_O_19_ content, while the BaTiO_3_ content remained constant. Morphological and structural characterization was performed in all nanocomposite systems via XRD patterns and SEM images, which verified that all specimens were successfully fabricated with a fine dispersion of inclusions. The recorded dielectric relaxations are related to both the polymer matrix (glass-to-rubber transition and re-orientation of polar-side groups) and the presence of reinforcing phases (IP). Dielectric permittivity increases with ceramic filler and diminishes rapidly with frequency. Nanocomposites with high SrFe_12_O_19_ filler loadings (i.e., 40 and 50 phr) exhibit an intermediate relaxation process called IDE, which is attributed to intrinsic interfacial polarization phenomena within SrFe_12_O_19_ grains. Dielectric response is affected by the occurring interactions between the constituents of the nanocomposites. From the analysis of the dynamics of the recorded relaxations (α-, β-relaxation, and IDE), activation energy and the VFT parameters were determined. The presence of strontium ferrite nanoparticles induces magnetic properties in the nanocomposites, which advance with SrFe_12_O_19_ content. Thermal stability and both static and dynamic mechanical response increase with the employed reinforcing phases, also supporting the conclusions from the morphological and dielectric studies for fine particle dispersion and good adhesion between ceramic particles and the polymer matrix. Finally, the fabricated nanocomposites can be employed for storing and retrieving electrical energy, and their ability increases with filler content.

## Figures and Tables

**Table 1:** Parameters evaluated via fitting data with Equations (3) and (4) for α- and β-process.

Composites	α-Mode		β-Mode *E_A_* (eV)
	*T*_0_ (K)	*A*	
Epoxy	321.47	18.21	1.256
5 phr SrFe_12_O_19_ + 10 phr BaTiO_3_	315.28	19.10	0.910
10 phr SrFe_12_O_19_ + 10 phr BaTiO_3_	313.98	19.51	0.888
15 phr SrFe_12_O_19_ + 10 phr BaTiO_3_	321.74	18.34	1.029
20 phr SrFe_12_O_19_ + 10 phr BaTiO_3_	314.60	18.42	1.765
40 phr SrFe_12_O_19_ + 10 phr BaTiO_3_	321.10	16.52	0.900
50 phr SrFe_12_O_19_ + 10 phr BaTiO_3_	317.26	15.17	2.569

## Data Availability

Data are available upon reasonable request.

## Supplementary Materials

The following supporting information can be downloaded at: https://www.mdpi.com/article/10.3390/polym14224817/s1, Figure S1: Example of Havriliak–Negami fitting process for β-relaxation for the 40 phr SrFe_12_O_19_/10 phr BaTiO_3_/epoxy nanocomposite at 130 °C; Figure S2: SEM image of the nanocomposite with 5 phr SrFe_12_O_19_/10 phr BaTiO_3_/epoxy, at a lower magnification; Figure S3: Representative stress-strain curves of all studied systems; Figure S4: TGA thermographs of all studied systems; Table S1: Glass transition temperatures determined via DSC, temperatures where the 5% mass loss occurs, and magnetic susceptibility of all studied systems.
