# Supplementary material for: Multifunctional Performance of Hybrid SrFe12O19/BaTiO3/Epoxy Resin Nanocomposites
Source: Polymers (Basel). 2022 Nov 9;14(22):4817. doi: 10.3390/polym14224817 (PMC9696728; doi:10.3390/polym14224817)
Supplement: Supplementary file 1 [file polymers-14-04817-s001.zip › polymers-1968935-supplementary.pdf]

Supplementary Material

Multifunctional Performance of Hybrid $SrFe_{12}O_{19}/BaTiO_3$/Epoxy Resin Nanocomposites

Georgia C. Manika[1]*, Sevasti Gioti[1], Aikaterini Sanida[1], Georgios N. Mathioudakis[2], Anxhela Abazi[1,3], Thanassis Speliotis[3], Anastasios C. Patsidis[1], and Georgios C. Psarras[1]*

[1]Smart Materials & Nanodielectrics Laboratory, Department of Materials Science, School of Natural Sciences, University of Patras, 26504 Patras, Greece

[2]Institute of Chemical Engineering Sciences (ICE-HT), Foundation for Research & Technology-Hellas (FORTH), Stadiou Str., Platani, P.O.Box 1414, GR-26504 Patras, Greece

[3]Institute of Nanoscience and Nanotechnology, NCSR "Demokritos", Aghia Paraskevi, 15310 Athens, Greece

*Correspondence: manikage@upatras.gr; Tel.: (+30 2610 997835) (G.C.M.); G.C.Psarras@upatras.gr; Tel.: (+30 2610 996316) (G.C.P.)

Supplementary Information

Fitting example for the determination of β-relaxation process by using the Havriliak Negami (HN) Equation (S1):

$$\varepsilon^*_{HN}(\omega) = \varepsilon_\infty + \sum_{k=1}^{2} \frac{\Delta\varepsilon_k}{[1+(i\omega\tau_{HN,k})^{a_k}]^{\beta_k}} - (\frac{\sigma_o}{i\varepsilon_0\omega})^N \quad \text{(S1)}$$

where k indicates the studied process, $\Delta\varepsilon_k(T)$ is the relaxation strength, σ_0 is the DC conductivity and N is the exponent which varies according to the conduction process. α and β parameters characterize the width and the symmetry of the loss peak. $\tau_{HN,k}$ is the Havriliak-Negami relaxation time and is related to τ_{max} with the following Equation (S2):

$$\tau_{max} = \tau_{HN}[\frac{\sin\left(\frac{\pi\alpha\beta}{2(\beta+1)}\right)}{sin\left(\frac{\pi\alpha}{2(\beta+1)}\right)}]^{1/\alpha} \quad \text{(S2)}$$

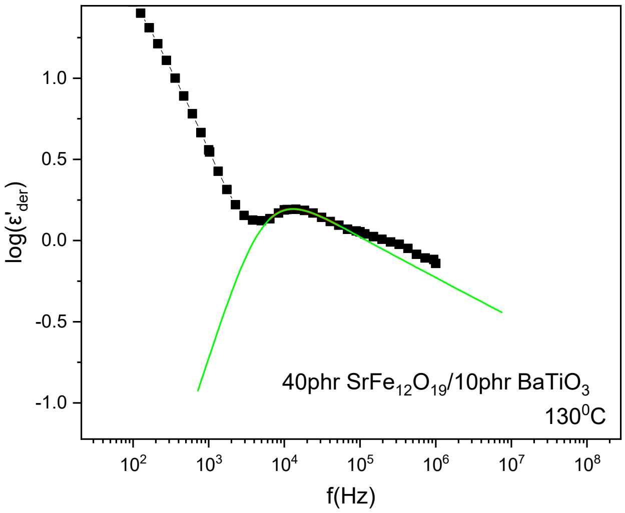

Figure S1. Example of Havriliak-Negami fitting process for β-relaxation for the 40phr $SrFe_{12}O_{19}$/10phr $BaTiO_3$/epoxy nanocomposite at 130°C.

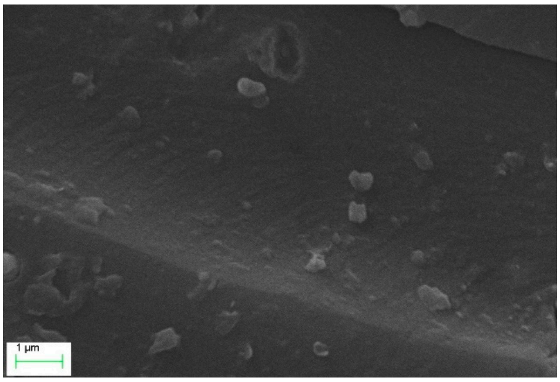

Figure S2. SEM image for the 5phr $SrFe_{12}O_{19}$/10phr $BaTiO_3$/epoxy at a lower magnification.

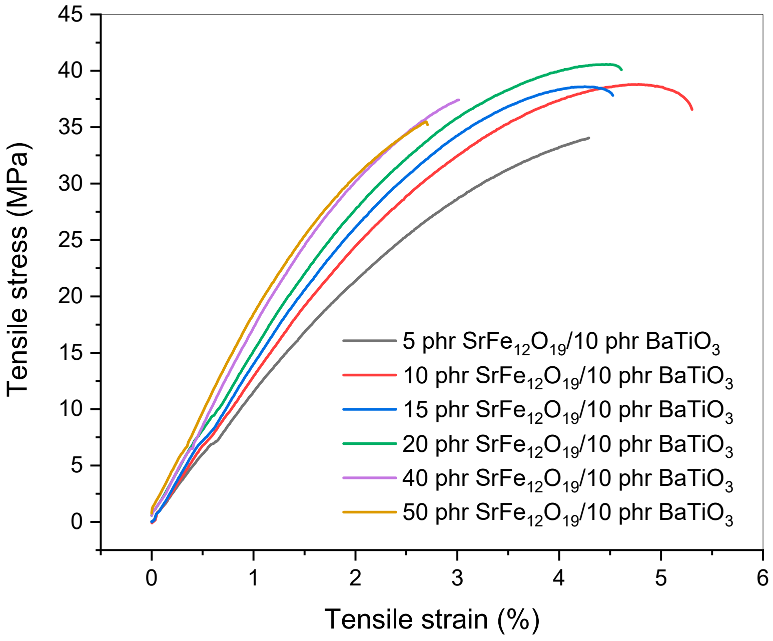

Figure S3. Representative stress-strain curves of all studied systems.

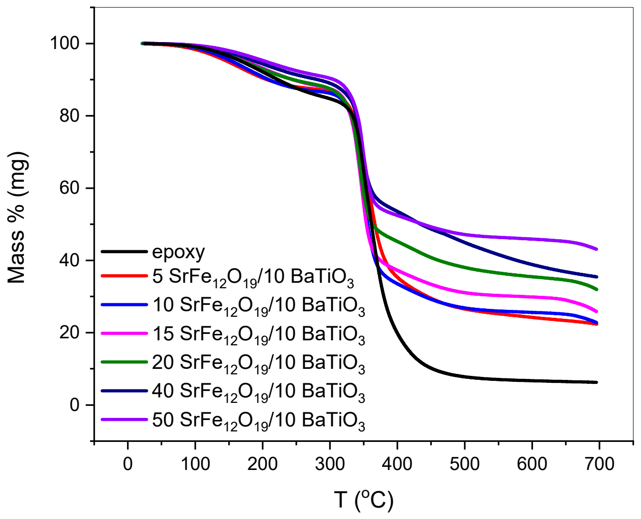

Figure S4. TGA thermographs of all studied systems.

Table S1. Glass transition temperatures determined via DSC, temperatures where the 5% mass loss occurs, and magnetic susceptibility of all studied systems.

**Composites**	**DSC** T_g **(°C)**	**TGA** ***T*** **of 5% mass loss (°C)**	**Magnetic susceptibility** x_m
Epoxy	48.13	169.17	-
5phr $SrFe_{12}O_{19}$ +10phr $BaTiO_3$	48.01	148.59	0.011
10phr $SrFe_{12}O_{19}$ +10phr $BaTiO_3$	51.78	155.50	0.021
15phr $SrFe_{12}O_{19}$ +10phr $BaTiO_3$	48.28	176.20	0.022
20phr $SrFe_{12}O_{19}$ +10phr $BaTiO_3$	47.46	172.40	0.051
40phr $SrFe_{12}O_{19}$ +10phr $BaTiO_3$	48.17	190.64	0.081
50phr $SrFe_{12}O_{19}$ +10phr $BaTiO_3$	47.25	205.07	0.090
